# Social norms, misperceptions, and mosquito net use: a population-based, cross-sectional study in rural Uganda

**DOI:** 10.1186/s12936-019-2798-7

**Published:** 2019-06-03

**Authors:** Jessica M. Perkins, Paul Krezanoski, Sae Takada, Bernard Kakuhikire, Vincent Batwala, Alexander C. Tsai, Nicholas A. Christakis, David R. Bangsberg

**Affiliations:** 1 Department of Human and Organizational Development, Peabody College, Vanderbilt University, Nashville, TN 37203 USA; 2 Vanderbilt Institute of Global Health, Vanderbilt University Medical Center, Nashville, TN 37203 USA; 3 Department of Medicine, University of California San Francisco, San Francisco, USA; 4 National Clinician Scholars Program UCLA, Division of General Internal Medicine and Health Services Research, Department of Medicine, University of California Los Angeles, Los Angeles, USA; 5 VA HSR&D Center for the Study of Healthcare Innovation, Implementation, & Policy, San Diego, CA USA; 6 Mbarara University of Science and Technology, Mbarara, Uganda; 7 Chester M. Pierce, MD Division of Global Psychiatry, Massachusetts General Hospital, Boston, USA; 8 Yale Institute of Network Sciences, Yale University, New Haven, USA; 9 Oregon Health & Science University, Portland State University School of Public Health, Portland, OR USA

**Keywords:** Malaria, Bed net, ITN, Perceived norm, Descriptive norm, Social norms, Peer norm, Misperception, Uganda

## Abstract

**Background:**

Mosquito net use is an essential part of malaria prevention. Although previous research has shown that many people sleep under a mosquito net in endemic areas, it is unknown whether people underestimate how common it is to sleep under a net every night. Furthermore, perceived social norms about whether most others sleep under a mosquito net every night may contribute to personally sleeping under a net, given decades of research showing that people often mimic others’ behaviours.

**Methods:**

Population-based data were collected from 1669 adults across eight villages in one rural parish in southwestern Uganda. Individuals’ perception about whether most adults in their community sleep under a mosquito net every night was compared with whether daily mosquito net use was the actual norm in their community to identify the extent of norm misperception. The association between whether an individual perceived daily mosquito net use to be the norm and personal mosquito net use was assessed while adjusting for the ratio of nets:people in the household and other factors.

**Results:**

Although the majority (65%) of participants reported sleeping under a mosquito net every night (and 75% did so among the 86% of people with at least one net), one-quarter of participants thought that most adults in their community did not sleep under a mosquito net every night. Another 8% were unsure how many nights per week most adults in their community sleep under a mosquito net. Participants who perceived that daily mosquito net use was the norm were 2.94 times more likely to report personally sleeping under a mosquito net every night (95% CI 2.09–4.14, p < 0.001) compared to participants who thought doing so was not normative, adjusting for other factors.

**Conclusions:**

Results suggest an opportunity for anti-malarial interventions to reduce misperceptions about mosquito net use norms and emphasize the commonness of daily mosquito net use in malaria-endemic regions. If people correctly perceive most others to sleep under a net every night, then they may personally do so when possible and support others to do so too.

## Background

Malaria is a leading cause of morbidity and mortality in the world. To prevent malaria transmission, the World Health Organization (WHO) recommends sleeping under a mosquito net on a daily basis for the 3.2 billion people worldwide who remain at risk of malaria [1]. Despite evidence that nets are effective in preventing malaria transmission and that most people have a net under which to sleep, some individuals still do not sleep under a mosquito net [2, 3].

Past research on protective behaviours and attitudes has found that many people underestimate the prevalence of a protective behaviour or attitude in a reference group and believe it to rarely occur or be held (i.e., they perceive a minority to do it or hold that belief) even when the protective behaviour or attitude is present among a majority in that reference group [4–9]. Likewise, people often overestimate the prevalence of risky behaviours and believe them to be common when they are not [10–21]. That is, perceived descriptive norms, which are the behaviours an individual believes to be the most common within a reference group [22], may not be actual descriptive norms (also known as collective norms), which are the behaviours actually engaged in by a majority of the reference group [23–26].

A similar phenomenon may occur with mosquito net use whereby people underestimate use among peers. For example, if people do not talk about sleeping habits with peers, then they may simply lack information about typical mosquito net use behaviour. Furthermore, if sleeping under a mosquito net is only discussed when someone has malaria symptoms, when mosquitoes have been noticed, or when there is a problem with a net, then using a mosquito net may seem less common than it is in reality. Similarly, local media (e.g., billboards, newspapers, television, radio) may disproportionately display or discuss people who are sick with malaria rather than people who are healthy, which also makes the protective behaviour seem less common. Thus, it is plausible that even in contexts where more than 50% of people sleep under a mosquito net every night, there may be many people who do not perceive this behaviour to be normative.

## Conceptual framework

Humans have a tendency to follow the herd. Misperceiving a protective behaviour as uncommon when the behaviour actually is common is problematic if people’s choices and actions are motivated by their perception of what is typical behaviour. Indeed, the classic sociological dictum suggests that: ‘what is perceived as real is real in its consequences’ [27]. In addition, decades of research in social psychology have demonstrated this conformity to social norms. People adopt behaviours even when the behaviour goes against what an individual would otherwise do and conform to what they perceive is accepted by others [28–31]. In general, humans’ desire to avoid being viewed as different or as an outcast and seek to avoid being socially sanctioned [32]. Thus, social norms, and more specifically, perceived social norms, may in part drive personally sleeping under a mosquito net every night.

The Theory of Planned Behaviour and the subsequent Integrated Behavioural Model conceptualize perceived norms (also labelled as subjective norms) as an important antecedent to intentions and behaviours [33–35]. Starting from these psychological frameworks, Social Norms Theory then applies a sociological lens to the role of social norms in predicting behaviour by identifying both perceived norms at the micro level (i.e., an individual’s perceptions about typical behaviour among a reference group) and actual norms at a meso level (i.e., actual typical behaviour among reference groups) as potentially important predictors of attitudes and behaviours [23]. This theory emphasizes separate measurement of these social norm constructs to allow for identification of norm misperception and to be able to assess the extent to which perceived norms predict behaviour above and beyond the predictive contribution of actual norms. The Social Norms Approach applies this theory to behavioural research and intervention by calculating and describing the extent of norm misperceptions and then, assuming misperceptions exist, designing messages around positive actual norms as the basis of a social norms intervention to change misperceived norms and ultimately change attitudes and increase healthy or positive behaviour [23, 26, 36–38]. This intervention approach focuses on existing positive norms among a peer group or community as opposed to showing negative messages, which may make harmful behaviour seem more common than is true and inadvertently increase the harmful behaviour. Additional theoretical discussion from the communication discipline builds upon these theories by focusing on moderators of the relationship between perceived norms and personal attitudes and behaviour [24, 39–41].

A large body of observational studies on topics of public health importance provides empirical support for these theories and frameworks by finding strong associations between perceived norms and personal behaviour (e.g., see these reviews for some examples [42–44]). In addition, studies using longitudinal analyses, quasi-experimental designs, and randomized controlled trials have shown that changes in individuals’ perceived norms about a variety of risky behaviours determine, at least in part, whether the individual personally engages in the behaviour [18, 21, 45–63]. Given this theoretical and empirical history, several recent review articles discuss the importance of changing perceived social norms (i.e., correcting misperceived norms) to change behaviour and also discuss issues to consider as part of developing interventions that try to change perceived norms by directly or indirectly providing social norms messages based on actual norms [44, 64–67]. Moreover, social norms messaging has been utilized within recent behavioural science-based interventions as an effective form of social nudging [68].

Although most of this work has primarily been conducted in high-income countries, a few studies from sub-Saharan Africa have identified misperceptions of social norms and associations between norm perception and personal behaviour about HIV-prevention related behaviours [9, 69–72] and environmental-related attitudes and behaviours [73]. In addition, a few experimental studies on other topics in Africa have found that changes in perceived norms were associated with changes in personal behaviour [74–77]. However, no studies on mosquito net use have compared the gap between an individual’s perception about whether daily mosquito net use was normative in a given population and the actual norm for mosquito net use in that population. If there are people who misperceive an existing positive mosquito net use norm, then an opportunity to correct the misperception is presented. And, if perception is associated with personal behavior, then intervening on individuals or a population to correct norm misperceptions might then increase daily mosquito net use among people who do not sleep under mosquito nets every night. In addition, people who sleep under a mosquito net for other reasons but who had misperceived the norm (and thus were ‘carriers of the misperception’) may feel more supported to continue engaging in the protective behaviour upon learning the true norm. They may also be less likely to spread incorrect assumptions about normative behaviour in their community during general conversation and support an overall climate of using mosquito nets.

## Current study

In Uganda, the vast majority of households now own mosquito nets due to a nationwide campaign that was conducted in 2013–2014 to ensure universal free access to mosquito nets and provide a ratio of at least one net for every two people in a household [78]. Yet, ownership has not translated into consistent use: according to 2014–2015 nationally representative data, one-quarter of people in Uganda with at least one net at home report not having slept under it the previous night [79]. Thus, a cross-sectional, population-based study on social norms and mosquito net use was conducted in rural Uganda. The study aimed to (1) quantify the prevalence of people who misperceived the daily mosquito net use norm; and, (2) determine the extent to which perception of the norm was associated with personal daily mosquito net use. It was hypothesized that a substantial amount of people would erroneously perceive that daily mosquito net use was not normative in their community despite most people sleeping under a mosquito net every night. The second hypothesis was that people who thought daily mosquito net use was normative would be much more likely to personally sleep under a mosquito net every night as compared to people who misperceived that most adults in their community did not do so.

## Methods

### Study population and procedure

The study was conducted in Nyakabare Parish, Mbarara District, a rural area of southwest Uganda. It is 20 km away from the nearest city, and is in a malaria-endemic region. The healthcare infrastructure is limited. Beginning in early 2011, the research team conducted a census enumeration of all adults (18 years+) who considered their permanent main household to be in the parish, which contained eight villages. The census was continually updated thereafter. For this population-based study, eligible participants were defined as all adults whose main household was within the parish. If there was more than one eligible adult in a household, then all such adults were eligible to participate in individual interviews.

Survey interview materials were iteratively translated and piloted from English to the local language and back to ensure accuracy and consistent word choice. The study team began contacting eligible participants in October 2011 for survey administration moving from village to village. Several well-trained research assistants who spoke the local language conducted one-on-one interviews lasting about an hour typically at the participant’s home. By the end of the data collection period in August 2012, 1669 adults had been interviewed, 16 refused, and 62 could not be contacted (because the person was always away). The response rate was 96%.

### Measures

#### Availability of mosquito nets in the household

All individual participants were first asked, “Does your household have a mosquito net that can be used while sleeping?” If the answer was yes, then further questions about mosquito net use were asked. Participants also reported the number of functional mosquito nets present in their household in response to the question, “In total, how many mosquito nets that can be used while sleeping do you have in your household?” as this factor is associated with use [80–84]. Combining this information with the total number of household members, a categorical variable representing the ratio of reported number mosquito nets to the number of people in the household was created to indicate access to a mosquito net within one’s household, with the following categories: 0 nets, ratio < 0.5, 0.5 to less than 1.0, 1.0 or greater.

#### Daily personal mosquito net use and the actual norm

If participants reported at least one mosquito net in their household, then participants were asked, “On average, how many nights per week do you sleep under a mosquito net?” A binary variable was created to capture whether a participant reported sleeping under a mosquito net seven nights per week. Given the population-based sample, the parish-wide prevalence of daily mosquito net use was calculated based on responses to the personal mosquito net use question. Daily mosquito net use was considered to be normative (i.e., to be the actual norm) if more than 50% of adults reported sleeping under a mosquito net seven nights per week.

#### Perceived norm for daily mosquito net use

To measure the perceived mosquito net use behaviour of most adults in their parish, participants were asked, “On average, how many nights per week do you think that most other people aged 18 years and older in your parish sleep under a mosquito net?” They could answer any digit from 0 to 7 or don’t know/unsure. This question was informed by previously published studies on other health-related risk behaviours [38, 85]. Based on responses, a trichotomous variable was created to indicate whether participants (a) thought that most adults sleep under a mosquito net seven nights per week (that is, engaged in daily mosquito net use); (b) thought that most adults do not sleep under a mosquito net seven nights per week (that is, engaging in less than daily use); or, (c) were unsure about how often most adults sleep under a mosquito net. This variable was created because this study was substantively focused on norms around daily use due to the malaria-endemic context. Participants in the first category perceived daily mosquito net use to be normative. Accuracy of norm perception was determined by comparing an individual’s perceived norm about daily net use in the parish to the actual norm about daily net use in the parish.

#### Other explanatory variables

Information on gender, age, marital status, education, household wealth, number of additional adults in the household (other than participant and spouse, if married), and number of children in the household was also collected because prior studies have identified associations between personal mosquito net use and these factors [83, 84, 86–92]. By including these variables, analyses could adjust for any potential confounding introduced by these factors on the relationship between perception and behaviour.

Age (15 missing) was a continuous measure. Marital status was categorized as married or single/separated/widowed/divorced (1 missing). Education (32 missing) was defined as having completed (a) none, (b) primary school, (c) secondary school, or (d) postgraduate studies. The number of household adults and children were continuous variables (82 and 81 missing, respectively). A household asset index was created to indicate wealth, by conducting a principal components analysis on 26 household assets and housing characteristics (no missing data). The first principal component was retained to define the wealth index and then split it into quintiles [93]. Finally, season was recorded using a binary variable to capture any variation due to wet versus dry season (that is March/April/October/November vs other months).

### Statistical analysis

The population is described and indicators of access to mosquito nets in the household are provided. Then, the prevalence of daily mosquito net use is shown across sub-groups as well as the percentage of people in each perception accuracy category across sub-groups. To test the relationship between perceived mosquito net use norm and personal use among people with at least one mosquito net in their household, we use a multivariable multilevel logistic regression model that accounts for the clustering of observations at the household level. Through this model, the log-odds of a participant reporting personal daily mosquito net use as a function of the participant’s perceived norm about daily mosquito net use was estimated adjusting for the nets:people household ratio, gender, marital status, age, number of additional household adults, number of household children, education, household wealth, season, and village (as dummy variables). All significance tests are conservative as the data represented the entire population.

## Results

Participant characteristics are presented in Table 1. More than 60% were under 40 years old, 58% were married, and 69% had completed primary education or less. The average number of adults per household was 1.8 (SD = 0.5). Eighty-six percent of participants personally reported having at least one mosquito net in their household. Overall, 68% of participants reported having enough mosquito nets in their household to indicate a ratio of one or more mosquito nets per every two people in their household (the national target). Forty percent of participants reported having three or more mosquito nets in their household.

**Table 1:** Prevalence of sleeping under a mosquito net among adults across eight villages in rural Uganda

Characteristics	n	%	% who sleep under a mosquito net every night	% who sleep under a mosquito net every night among people reporting at least one net in the household
Gender				
Male	913	54.7	61.7	77.2
Female	756	45.3	67.1	72.9
Age (years)				
Under 30	694	42.0	65.3	74.1
30–39	330	20.0	67.6	77.2
40–49	266	16.1	69.6	77.4
50–59	134	8.1	62.4	74.8
60–69	97	5.9	57.3	73.3
70–79	74	4.5	62.2	82.1
80 or more	59	3.6	42.1	64.9
Marital status				
Married	961	57.6	64.6	67.7
Single/divorced/separated	707	42.4	53.8	80.2
Education				
No education	276	16.9	56.6	73.7
Primary	847	51.7	64.6	75.2
Secondary	405	24.7	67.7	74.1
Postgraduate	109	6.7	74.3	86.2
Household asset quintile				
Lowest	273	16.4	55.3	73.3
2nd	316	18.9	64.2	74.4
3rd	327	19.6	70.8	80.4
4th	383	23.0	66.1	73.3
Highest	370	22.2	65.0	75.0
Ratio of nets:people in the household				
0 nets	235	14.1	0	n/a
< 0.5	291	17.4	54.5	54.5
0.5 to < 1.0	437	26.2	73.2	73.2
1.0 or greater	706	42.3	85.1	85.1

Daily mosquito net use among adults was the actual norm in this parish as 65% of all participants reported sleeping under a mosquito net every night. (This percentage incorporates participants who had no mosquito nets). Among participants reporting at least one mosquito net in the household, 75% reported sleeping under a net every night. Furthermore, sleeping under a mosquito net every night was also the actual norm for many sub-groups as 54–85% of people in different sociodemographic categories reported sleeping under a net every night (except for people aged 80 years or more and people without a net in the household) (Table 1). Even the majority of participants living in a household with fewer than one net for every two people (but at least one net in the household) reported sleeping under a mosquito net every night. Similarly, 57–81% of adults across each village reported doing so each night (Table 2).

**Table 2:** Prevalence of sleeping under a mosquito net every night across eight villages in rural Uganda

Village	n	%	Mean ratio of the number of mosquito nets per person in the household	% who sleep under a mosquito net every night	% who sleep under a mosquito net every night among people who reported at least one net in their household
1	230	13.8	0.75	56.5	71.0
2	263	15.8	0.86	65.7	76.8
3	209	12.5	0.98	80.9	87.6
4	214	12.8	0.77	56.6	68.6
5	153	9.2	0.98	73.0	79.9
6	237	14.2	0.76	62.9	71.0
7	146	8.8	0.81	63.2	72.8
8	217	13.0	0.63	61.3	74.3

Yet, 23% of participants erroneously thought that the majority of adults in their parish do not sleep under a mosquito net every night, and thus misperceived the norm. This level of norm misperception was consistent as 20–30% of participants across sociodemographic sub-categories believed that daily mosquito net use was not normative in the parish (Table 3).

**Table 3:** Prevalence of perceived norm accuracy about daily mosquito net use among adults in rural Uganda

Respondent characteristics	Accurate perception: % who thought that daily mosquito net use was normative in the parish	Erroneous perception: % who thought that daily mosquito net use was not normative in the parish	Did not know perception: % who insisted on not knowing how often people in their parish sleep under a mosquito net
Gender			
Male	67.6	23.8	8.6
Female	68.9	23.1	8.0
Age (years)			
Under 30	72.0	23.5	4.5
30–39	70.6	21.8	7.6
40–49	68.4	21.4	10.2
50–59	64.9	21.6	13.4
60–69	61.9	28.9	9.3
70–79	58.1	25.7	16.2
80 or more	45.8	28.8	25.4
Marital status			
Married	70.1	22.3	7.6
Single/divorced/separated	65.8	25.0	9.2
Education			
No education	63.0	21.0	15.9
Primary	68.0	24.7	7.3
Secondary	72.1	22.5	5.4
Postgraduate	70.6	22.9	6.4
Household asset quintile			
Lowest	61.5	24.9	13.6
2nd	72.8	21.2	6.0
3rd	68.2	23.9	8.0
4th	69.5	23.5	7.0
Highest	68.4	23.8	7.8
Ratio of nets:people in household			
0 nets	55.3	24.3	20.4
< 0.5	64.6	29.6	5.8
0.5 to < 1.0	72.3	21.1	6.6
1.0 or greater	71.7	22.1	6.2

Daily mosquito net use among adults was normative if more than 50% of participants in the parish personally reported sleeping under a mosquito net seven nights per week

For example, 24% of participants with zero nets in their household misperceived daily mosquito net use as uncommon in their parish as did 22% of participants in a household with a ratio of nets:people of 1.0 or greater. In addition, 8% of participants indicated not knowing how often adults in their parish sleep under a mosquito net. Thus, almost one-third of all participants did not realize that daily mosquito net use was the norm in their parish when combining participants who misperceived the norm with participants who were unsure about the norm. Similarly, 17–38% across villages misperceived the norm (Table 4).

**Table 4:** Prevalence of perceived norm accuracy about mosquito net use among adults in rural Uganda

Village	Accurate perception: % who thought that daily mosquito net use was normative in the parish	Erroneous perception: % who thought that daily mosquito net use was not normative in the parish	Did not know perception: % who insisted on not knowing how often people in their parish sleep under a mosquito net
1	70.4	23.5	6.1
2	70.3	22.1	7.6
3	76.6	17.2	6.2
4	62.6	19.6	17.8
5	56.9	37.9	5.2
6	72.2	20.3	7.6
7	58.9	32.9	8.2
8	71.4	21.7	6.9

Excluding participants who reported zero nets in their household, a simple bivariate association showed that among participants who perceived daily mosquito net use as normative, 81% reported personally sleeping under a mosquito net seven nights per week. In contrast among participants who thought daily mosquito net use was not normative, 61% reported personally sleeping under a mosquito net every night. Further, among participants who did not know their perception of the norm, 63% reported daily mosquito net use. Subsequent regression analyses found that an individual’s perception about the normative mosquito net use behaviour had a statistically significant association with personal mosquito net use after adjusting for several other explanatory variables (Table 5). Participants who perceived daily use as normative in their parish were 2.94 times more likely (95% CI 2.09–4.14, p < 0.001) to sleep under a mosquito net every night compared to participants who perceived that most adults in their parish did not do so while adjusting for several other variables. Other factors that were associated with daily mosquito net use included the ratio of nets:people in the household, the number of children in the household, the number of additional adults in the household, being female, being married, and having postgraduate education. For example, compared to having access to fewer than one net for every two people in the household, having a ratio of ‘0.5 to < 1.0’ nets per person in the household was associated with a 2.21 greater likelihood (95% CI 1.47–3.32, p < 0.001) of personally sleeping under a net every night. A supplemental analysis found similar estimates of the association between perception of the norm and personal use for both men and women.

**Table 5:** Odds ratios for personally sleeping under a mosquito net every night among adults in rural Uganda

	AOR	(95% CI)	*p* value
Perceived daily mosquito net use as normative	2.94	(2.09, 4.14)	< 0.001
Did not know perception	1.10	(0.60, 2.03)	0.760
Perceived daily mosquito net use as not normative	1.0	–	–
Nets:people in the household from 0.5 and < 1.0 (vs < 0.5)	2.21	(1.47, 3.32)	< 0.001
Nets:people in the household is 1.0 and greater (vs < 0.5)	4.48	(2.91, 6.90)	< 0.001
Female (vs male)	1.47	(1.09, 1.99)	0.012
Married (vs single)	1.87	(1.33, 2.63)	< 0.001
Age	1.00	(0.99, 1.01)	0.548
Number of children in the household	0.85	(0.78, 0.93)	< 0.001
Number of additional adults in the household	0.89	(0.81, 0.98)	0.023
Primary school (vs none)	0.97	(0.61, 1.56)	0.900
Secondary school (vs none)	1.18	(0.67, 2.09)	0.561
Postgraduate studies (vs none)	2.43	(1.06, 5.61)	0.037
2nd household wealth quintile (vs lowest)	1.09	(0.63, 1.87)	0.766
3rd household wealth quintile (vs lowest)	1.98	(1.11, 3.54)	0.021
4th household wealth quintile (vs lowest)	1.49	(0.86, 2.99)	0.158
Highest household wealth quintile (vs lowest)	1.65	(0.91, 1.38)	0.102
Rainy season	0.97	(0.67, 1.38)	0.848

Estimates were obtained using a multilevel logistic regression model that accounted for clustering of observations at the household level and also included dummy variables for village

## Discussion

In this study, 23% of adults in a malaria-endemic region misperceived sleeping under a mosquito net every night as rarely occurring among most adults in their community even though most adults personally reported sleeping under a mosquito net every night. Moreover, 5–25% of adults (depending on the sociodemographic category) indicated that they did not know how many nights per week most adults in their parish slept under a mosquito net. This novel identification of misperceived norms about regular mosquito net use is similar to results demonstrating the discrepancy between actual and perceived behavioural norms from social norms studies on other health-related behaviours and attitudes [4–7, 10–21, 25, 26, 65, 94–96]. In addition, underestimating the normativity of a protective behaviour is similar to results about misperception of HIV testing norms within this same population in rural Uganda [9] and about HIV prevention behaviour in Tanzania and South Africa [69, 70, 72].

The second novel contribution of this study was finding that perceiving daily mosquito net use to be normative was a strong protective factor for personally sleeping under a mosquito net every night. The finding that the perceived norm is strongly associated with personal behaviour is consistent with results from studies on other topics [18, 21, 45–58, 84, 97, 98]. In follow-up research, it will be important to assess whether malaria-specific knowledge and perceptions of risk, and belief in the effectiveness of mosquito nets to prevent malaria, confound or modify the association between perception and behaviour [84, 99–104].

### Intervention implications

The results of this study suggest that there is an opportunity to correct mosquito net use norm misperceptions by increasing adults’ awareness of daily mosquito net use among peers. A social norms approach to doing so would first create messages about the positive actual behavioural norms about mosquito net use among specific reference groups in this context. Thus, example fact-based messages might be ‘Most adults in this village sleep under a mosquito net every night’ or ‘Most single men in village X sleep under a mosquito net every night’. Highlighting the typical ratio of nets available for use within the household could also be a helpful message. For example, data from this study indicated that the ownership norm represented the national target; that is ‘Most people in your village have access to at least one net for every two people in their household’. Emphasizing true protective norms (e.g., most people use nets in X village) rather than publicizing the problem (e.g., the number of people suffering from malaria in a community) will be more effective in bringing about the desired behaviour change [105, 106]. In addition, messages need to be credible, for example, created from recent local data [67, 106–108].

Interventions disseminating social norms messages have typically been implemented as community-wide social norms marketing campaigns, personalized normative feedback sessions, or facilitator-led small group discussions about actual norms and norm misperceptions [64]. However, the specific design of a social norms intervention should be tailored to the community setting. For example, a community-wide social norms marketing intervention in this kind of context could disseminate information on true behavioural norms about mosquito net use among specific reference groups using a variety of communication methods such as billboards, radio shows, education-entertainment, or SMS text-messages. Alternatively, local leaders or community health workers could provide information on actual norms about mosquito net use during local group meetings and engage in community-based dialogues about these norms. Separately, they could provide personalized normative feedback during one-on-one conversations. Although the delivery mode in a given context to a specific population should be assessed for feasibility and acceptability, a combination of communication approaches would likely be most impactful [109], especially as doing so increases frequency of, and thus exposure to, messaging.

This kind of intervention messaging may integrate well with other malaria-related behaviour change communication programmes [110]. Actual norms messaging could also increase the impact of structural anti-malaria interventions by creating a more informed population to target or with whom to operate. For example, messages about the commonness of net ownership and net use could be paired with a campaign to distribute free mosquito nets, potentially motivating more people to pick up and use a net regularly. These kinds of low-cost social norms-based interventions may have great utility in settings such as sub-Saharan Africa [111]. However, development of an intervention using a social norms approach to improve mosquito net use should account for other local factors that may influence personally sleeping under a net every night [112]. In addition, a norms-based intervention to increase mosquito net use should not be conducted alongside interventions that call attention to extreme cases of malaria or foster fear as they mute effects from any social norms messaging by implying that people are not using nets [106, 107, 113].

For individuals who misperceive the norm before a social norms intervention is implemented and who do not sleep under a mosquito net regularly, it is plausible that an intervention to correct misperceived norms may change their perception and therefore encourage them to conform to the normative behaviour and sleep under a net every night given the association between perception and behaviour. In addition, individuals who misperceive the norm but who do sleep under a net every night for other reasons may feel more supported to continue doing so every night upon learning that their behaviour is actually normative. Finally, individuals accurately perceiving the norm before the intervention may feel additionally supported by a social norms intervention, and therefore encouraged to vocally reinforce to others the importance and commonness of sleeping under a mosquito net every night.

## Study limitations

Interpretation of these findings is subject to limitations. First, the data are cross-sectional so causal direction between norm perception and personal behaviour cannot be determined. However, previous studies using longitudinal and experimental designs provide extensive evidence that change in an individual’s perception of the norm has led to changes in personal behaviour for several health-related behaviours (as cited previously).

A second limitation is that data about personal mosquito net use are based on self-report. Although the rates reported in this study are similar to rates from past research [79], it is possible that reporting may have been influenced by social desirability bias (i.e., given the regular government campaigns) or by their perceptions of the norm (whether correctly perceived or incorrectly perceived). While the extent of mis-reporting is quantifiable and important, it is unclear whether the degree of mis-reporting could be significant enough to change the findings reported here. For example, a recent meta-analysis of studies comparing self-report and objective data found that people overestimated their mosquito net use by 13.6% [114]. Thus, even if 10–15% of participants in this study had incorrectly reported sleeping under a mosquito net seven nights per week when they actually sleep under a net less often, the majority of participants in this study would still be using a mosquito net every night. In that scenario, the actual norm would not have changed and norm misperception would still exist within this population. Moreover, as participants were asked how many nights they sleep under a mosquito net, mis-reports from participants who reported using a net less than seven nights per week but still over-estimated the number of nights they sleep under a mosquito net would not affect the results from this study as this study is discussing a binary norm about daily use. Finally, even if there were people whose under-reporting was driven by perceptions that others did not use nets, then the prevalence of actual use would be even greater and the extent of norm misperception found in this study would still exist.

## Future research directions

Future research on mosquito net use norms would improve if objective monitoring methods were used to measure actual net use norms [101]. In addition, follow-up studies could assess the importance of norms about different social reference groups [115–118]. Inquiring about norms about “men your age in your village” or “women with young children” could perhaps show less norm misperception. However, although the potential association between perceived norms about proximal peers and personal behaviour may be stronger than the association between perceived norms about distal peers and personal behaviour, the extent of close peer norm misperception, and thus the possible extent of perceived peer norm correction would likely be less [26]. In contrast, even though the perceived norm about distal peers may be less influential, there is likely to be more norm misperception about distal peer groups [116]. This greater misperception thus allows for more potential change to occur in the perceived norm, and ultimately, perhaps, in personal behaviour. Relatedly, collecting network data could examine whether clustering of perceived norms about mosquito net use and actual use behaviours occurs within friendship networks or other kinds of networks [119]. Social network structure and the clustering of specific behaviour among close friends might make that behaviour seem more common overall than it actually is in the larger reference group [120].

Assessing social norms about a variety of behaviours related to mosquito net use when malaria vectors are most present, such as entry and exit of nets at night or the early morning, may also be worthwhile [121, 122]. Likewise, the power of perceptions about societal expectations around mosquito net use may also play a role. Injunctive norms, that is perceptions about what people ought to do, are distinct from behavioural descriptive norms and can exert their own direct influence or a modifying influence on the relationship between descriptive norms and personal behaviour [22, 24, 123].

## Conclusions

There are two main findings from this population-based study on mosquito net use in rural Uganda. First, even though daily mosquito net use was reported by a majority of people in the targeted community, about a quarter of people misperceived daily use as not normative in this population, and another 8% did not know whether it was normative. Second, people who thought daily mosquito net use was normative among adults in their community were much more likely to report personally sleeping under a mosquito net every night. The estimated association was statistically significant, large in magnitude, and robust. These findings suggest an opportunity for anti-malarial interventions to correct misperceived norms about daily mosquito net use in malaria-endemic regions. An increase in accurately perceiving this protective behaviour to be common and normative may help advance malaria prevention efforts in sub-Saharan Africa.

## Data Availability

The datasets used and/or analysed during the current study are available from the corresponding author on reasonable request.
